# Prospective evaluation of acute side effects profiles in moderately hypofractionated whole-pelvic radiotherapy for prostate cancer

**DOI:** 10.1016/j.ctro.2026.101169

**Published:** 2026-04-23

**Authors:** Leo Andexlinger, Barbara Knäusl, Andreas Renner, Maximilian Schmid, Dietmar Georg, Joachim Widder, Gregor Goldner, Gerd Heilemann

**Affiliations:** a Department of Radiation Oncology, Comprehensive Cancer Center Vienna, Medical University of Vienna, Währinger Gürtel 18-20, Vienna, 1090, Austria; b Christian Doppler Laboratory for Image and Knowledge Driven Precision Radiation Oncology, Department of Radiation Oncology, Medical University of Vienna, Währinger Gürtel 18-20, Vienna, 1090, Austria

**Keywords:** Patient-reported outcomes, Prostate cancer, Moderately hypofractionated radiotherapy, Whole-pelvic radiotherapy

## Abstract

**Background:**

Outcomes of whole-pelvic radiotherapy (WPRT) in the era of moderately hypofractionated radiotherapy for prostate cancer remain sparsely reported. Previous studies indicate a high discrepancy between patient-reported outcomes (PROs) and clinician-reported outcomes (CROs) assessing the same endpoint. This study analyzed the concordance between PROs and CROs for WPRT and prostate-only radiotherapy (PRT) patients.

**Methods:**

Patients received a prescribed dose of 20 × 3 Gy to the planning target volume (PTV) and, in cases of lymph node involvement, 46 Gy to the regional lymph nodes. 176 WPRT and 62 PRT patients reported genitourinary (GU) and gastrointestinal (GI) adverse events through an electronic patient-reported outcome measures tool. Simultaneously, clinicians graded side effects using Radiation Therapy Oncology Group (RTOG) criteria for up to two years post-radiotherapy. Composite GU and GI PRO scores, derived by averaging endpoints, were evaluated for their ability to discriminate RTOG *G1+* events via receiver operating characteristic curve analysis.

**Results:**

During treatment, WPRT patients exhibited elevated GI scores compared to PRT patients via PROs (mean: 2.80 vs. 2.48, p=0.010) and CROs (mean: 1.15 vs. 0.89, p=0.014), though levels equalized by 12 months. Composite PRO scores demonstrated moderate-to-good discriminatory performance for elevated CRO levels (areas under the curves: GI 0.65-0.74; GU 0.73-0.82) from the acute phase through 24 months.

**Conclusion:**

WPRT was associated with increased acute GI adverse effects but showed recovery to PRT levels within one year. Results demonstrated that PROs yield clinically relevant discriminatory value. Routine integration of PROs may facilitates earlier adverse event identification, allocation of clinical resources, and individualized clinical decision-making.

## Highlights

• Whole-pelvis radiation therapy outcomes reverted to prostate-only levels within one year.
• Composite side effect scores were derived from patient-reported symptoms.
• Patient-reported scores predicted RTOG ≥ 1 events.
• Routine collection of patient-reported outcomes facilitates resource allocation.

## 1 Introduction

External beam radiotherapy (EBRT) is a cornerstone of curative-intent treatment for primary prostate cancer (PCa) and administered in approximately 30% of patients [1]. By delivering higher dose per fraction in fewer treatments, hypofractionation (HF) results in fewer hospital visits, increasing patient throughput and reducing treatment cost [2]. Large clinical trials confirmed that moderately hypofractionated radiotherapy (MHRT) is non-inferior to conventional fractionation for treating PCa. While some studies reported a transient increase in acute radiation-induced gastrointestinal (GI) symptoms with MHRT, prevalence of genitourinary (GU) adverse side effects was generally similar, solidifying MHRT as a standard of care [3], [4], [5], [6], [7], [8].

Beyond prostate-only radiotherapy (PRT), whole-pelvic radiotherapy (WPRT) is prescribed for patients with nodal involvement (cN1) or high nodal risk. However, the symptom profiles of WPRT in the era of MHRT remains controversial. While the POP-RT trial reported increased cumulative late GU toxicity with WPRT following dose-escalated MHRT [9], other studies such as the PIVOTAL trial observed no significant differences after conventional fractionation [10]. Ongoing trials including PIVOTALboost and PEACE 2 aim to clarify these discrepancies [11], [12].

As overall survival of PCa patients continues to improve [13], focus has increasingly shifted toward long-term quality of life. Despite advances in radiotherapy, up to 30% of patients experience moderate or severe late side effects [14]. Adverse events are traditionally assessed using clinician-reported outcomes (CROs), e.g. based on Radiation Therapy Oncology Group (RTOG) criteria [15]. However, patient-reported outcomes (PROs) provide a complementary perspective by directly capturing symptom burden from the patient’s viewpoint [16]. The extent to which PROs reflect clinically relevant side effects, and correspond to clinician-assessed RTOG grades, remains insufficiently understood [17].

In this prospective real-world study, we analyzed the prevalence of GU and GI adverse side effects in PCa patients treated with a modern MHRT irradiation schedule. We specifically compared outcomes between WPRT and PRT cohorts. A further aim was to investigate the sensitivity and specificity of PROs as a surrogate for their clinician-reported RTOG counterparts.

## 2 Methods

### 2.1 Patient cohort

Within the prospective Patient Experience Data in Radiation Oncology (PEDRO) trial (NCT05224297) patient data on adverse side effects from patients receiving EBRT were collected between October 1, 2020 and May 5, 2025. The PEDRO trial was approved by the Institutional Review Board of the Medical University of Vienna. It is a prospective clinical trial collecting PRO data on adverse side effects from patients receiving EBRT.

All patients treated for PCa were eligible for participation after providing their informed consent which resulted in a total of n=507 patients. Inclusion criteria for this prospective evaluation study were at least one recorded PRO-questionnaire and RTOG score during the treatment course and follow-up period. Further patients needed to undergo MHRT treatment for primary PCa, and not having a prior surgical resection of the prostate, resulting in n=238 patients.

### 2.2 Treatment

Patients undergoing MHRT received 20 × 3 Gy to the prostate itself (prescribed to 95% of the PTV) and were classified into a low-, intermediate- and high-risk group. For low-risk patients the clinical target volume (CTV) was only comprised of the prostate while for intermediate-risk patients the CTV was extended to the base of the seminal bladder. By default all low and intermediate-risk patients received PRT. All high-risk patients and those with unfavorable intermediate-risk (defined as Roach score > 15% [18] or cN1) received WPRT. In these cases, the iliac lymph nodes were additionally simultaneously irradiated with 46 Gy. The CTV then included the external, internal, and common iliac lymph nodes up to the aortic bifurcation (L4/5).

All patients were treated in the supine position with full bladder and a rectal balloon was used for prostate immobilization during treatment. Safety margins around the CTV were 7 mm. All patients were treated with volumetric modulated arc therapy (VMAT) and androgen deprivation therapy (ADT) was prescribed at the discretion of the treating physician. After radiation therapy concluded, supportive measures for symptom management were continued or started.

**Table 1:** Patient demographic, clinical, and treatment characteristics, including age in years, clinical T stage, clinical N stage, Gleason score, maximal prostate-specific antigen (PSA) prior to radiotherapy (RT), administration of hormone therapy (hormone therapy (HT)), and risk group stratification.

	WPRT	n(%)	PRT	n(%)	p
n	176	–	62	–	
Age [years]					(T) 0.139
Median [min–max]	76.0 [55.0–88.0]		74.5 [54.0–85.0]		
Missing	0	(0)	0	(0)	
Clinical T Stage					($\chi^{2}$) < 0.001
T1a-T1b-T1c-T1x	103	(59)	54	(87)	
T2a-T2b-T2c-T2x	46	(26)	7	(11)	
T3a-T3b	23	(13)	1	(2)	
Missing	4	(2)	0	(0)	
Clinical N Stage					($\chi^{2}$) = 0.014
N0	69	(39)	15	(24)	
N1	13	(7)	0	(0)	
Nx	92	(52)	47	(76)	
Missing	2	(1)	0	(0)	
Gleason Score					($\chi^{2}$) < 0.001
6	4	(2)	23	(37)	
7	99	(56)	39	(63)	
8–10	73	(41)	0	(0)	
Missing	0	(0)	0	(0)	
Maximal PSA before RT [ng/ml]					(T) < 0.001
Median [min–max]	10.3 [3.3-190.0]		6.2 [1.5–18.3]		
Missing	0	(0)	0	(0)	
HT					($\chi^{2}$) < 0.001
Yes	107	(61)	16	(26)	
No	62	(35)	43	(69)	
Missing	7	(4)	3	(5)	
Risk Group					($\chi^{2}$) < 0.001
Low	0	(0)	20	(32)	
Intermediate	73	(41)	42	(68)	
High	103	(59)	0	(0)	
Missing	0	(0)	0	(0)	

### 2.3 Adverse side effect assessment

CROs, namely GI and GU adverse radiation-induced side effects, were reported as the maximal assessed RTOG score (treatmax) [15] by the treating radiation oncologist during treatment and at scheduled follow-up appointments, which took place at 3 and 12 months after treatment concluded. Subsequent follow-up visits were scheduled at one-year intervals. Follow-up sessions involved an assessment of symptoms, the PSA level, status of hormone therapy and, in case of bleeding, a rectoscopy.

Complementary, patients completed predefined electronic patient-reported outcome measures (ePROM) questionnaires [19] at the day of the first treatment session (baseline), at one-week intervals during treatment and at their last treatment session (lastfx). Follow-up ePROM assessment was conducted from home at 3, 6, and 12 months, and then annually after treatment completion. Questionnaires included Patient-Reported Outcomes version of the Common Terminology Criteria for Adverse Events (PRO-CTCAE) items [20] with an additional endpoint added based on institute-specific clinical observations. Sub-questions of endpoints assessed either the frequency of occurrence, severity, or interference with life of symptoms and are detailed in Supplementary Table S1.

To quantify the overall GI and GU symptom burden experienced by patients, a composite PRO score was computed [21], [22]. For each assessed endpoint, the maximum score across sub-questions was determined; these maxima were then averaged to obtain the mean composite score for GI and GU adverse side effects. The composite PRO scores consisted of the following endpoints: bloating, constipation, and diarrhea for GI side effects; and painful urination, urinary urgency, urinary frequency, and weak urine stream for GU side effects, respectively. Note that PRO scores assess the maximum symptom intensity experienced in the last seven days, whereas CRO scores represent the maximum clinically relevant adverse effects that occurred since the last assessment.

To conduct a similar comparison of PRO scores between cohorts during the treatment phase, we chose to report the distribution of the maximum composite PRO scores across all treatment weeks (treatmax), similar to the maximal RTOG grade. Furthermore, for individual PRO endpoints, we report the change-from-baseline (score minus baseline score) during treatment, after treatment (lastfx), and at each follow-up time point.

### 2.4 Statistical analysis

Descriptive statistics were used to summarize clinical characteristics of the study cohort (Table 1) including questionnaire response rates. Continuous variables, all assumed to be normally distributed, are presented as medians and compared using the Student’s T test. Categorical variables are presented as counts and percentages and were compared using the $\chi^{2}$ test.

To assess non-responder bias, we compared CRO distributions at each follow-up time point between patients who submitted PRO questionnaires and those who did not. Differences in distributions were statistically assessed by the Mann–Whitney U test at each time point.

Distributions of individual change-from-baseline PRO scores between WPRT and PRT cohorts were compared with the Mann–Whitney U test at treatmax, 3 months (3 m), 12 months (12 m), and 24 months (24 m). Longitudinal changes from baseline in the side effect profile were identified using the Wilcoxon’s signed-rank test for all time points (lastfx, 3 m, 12 m, 24 m).

Since the composite PRO score was constructed as the mean of multiple endpoints, it may take non-integer values. For visualization, the results were binned, but original values were used for calculating the distributions mean and 95% confidence interval (CI) according to the T-distribution. In analogy to prior individual analysis, the distributions of composite PRO and CRO scores were compared between WPRT and PRT cohorts with the Mann–Whitney U test (treatmax, 3 m, 12 m, 24 m).

Additionally, we evaluated the efficacy of using PROs as a surrogate for the detection of clinically relevant CRO events with receiver operating characteristic (ROC) curve analysis. Events were defined by the characteristic function in Eq. (1): A patient i was considered to have experienced a PRO or CRO grade ≥T event at time point $t_{j}\in \left\{\text{treatmax},\text{3m},\text{12m},\text{24m}\right\}$, if the corresponding score $S_{C}\left(i,t_{j}\right)$ exceeded the threshold T, where C represents one of the classes GI or GU.

(1) $$E_{C}\left(i,t_{j},T\right)=\left\{\begin{array}{c} 1,\text{if}S_{C}\left(i,t_{j}\right)\geq T\;\\ 0,\text{if}S_{C}\left(i,t_{j}\right)<T\; \end{array}\right)$$

Although grade ≥2 events (*G2+*) are commonly reported in the literature [9], [10], we defined clinically relevant CRO events at a lower threshold ($T_{CRO}=1$) to increase event numbers and to capture the first measurable deviation from baseline. ROC curve analysis was performed, by varying $T_{PRO}$ over its range for two principal objectives. Firstly, the concordance of PRO and CRO events at each time point was evaluated. Secondly, optimal event-classification thresholds for composite PRO scores $T_{PRO}$ were determined for each time point separately by the Youden-Index, maximizing the discrepancy between true and false positives. For all ROC curves the area under the curve (AUC) was reported. 95% CIs of AUC and thresholds were estimated by repeating the ROC analysis resampling the data with replacement 1000 times. Additionally, an empirical p-value was derived from the bootstraped distributions. The p-value represents the proportion of the 1000 AUCs that are lower than 0.5, which corresponds to the null hypothesis $\left(H_{0}\right)$ of no discriminative ability (AUC = 0.5).

All tests were two-sided with a significance level of α=0.05. For multiple testing, Holm–Bonferroni correction was applied ($\alpha_{i}=\frac{0.05}{n-i+1}$, where n is the number of tests). All analyses were performed in Python. For data processing and statistical analysis we used NumPy 1.26.1 and SciPy 1.11.3, scikit-learn 1.3.2. Visualization was performed with Plotly 5.9.0.

## 3 Results

### 3.1 Patient cohort

**Fig. 1:**
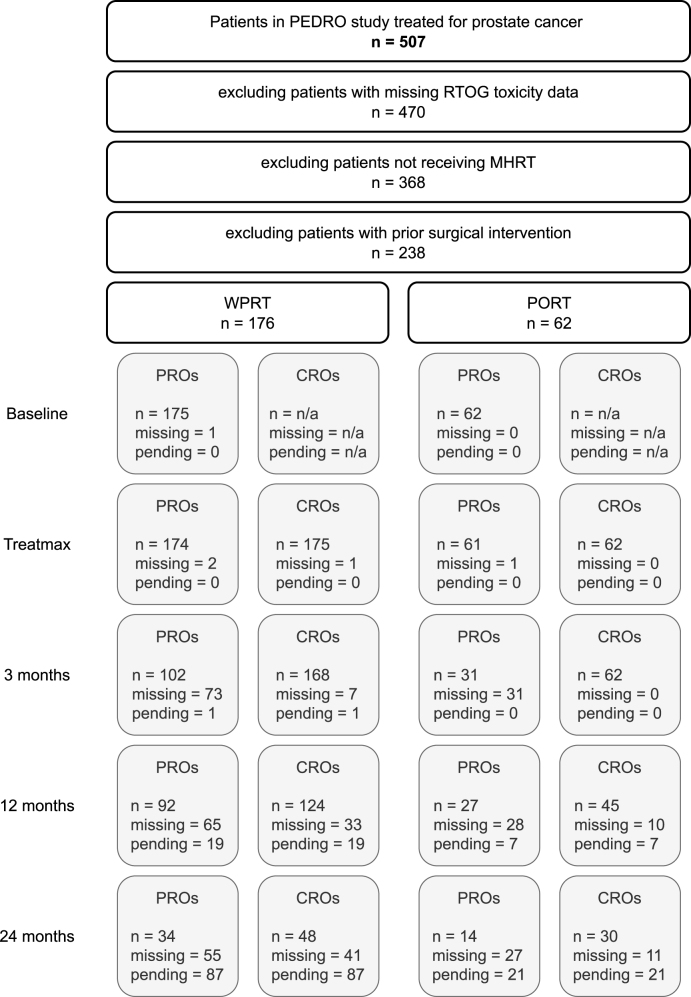
Depiction of patient selection process criteria and data completeness. The initial cohort comprised patients treated for prostate cancer within the PEDRO study. Exclusions were applied for missing RTOG data (n=37), deviation from the moderately hypofractionated radiotherapy fractionation schedule (n=102), or prior surgical intervention (n=130).

A breakdown of the patient selection process is depicted in Fig. 1. The final study cohort consisted of 238 patients (176 WPRT and 62 PRT).

Response rate of PRO questionnaires was high during treatment (≥95.0%), but rapidly decreased to 56.1% at the first 3-months follow-up. Response rates remained at equivalent level of 56.1% at 12 months, but subsequently deteriorated to 36.9% at 24 months. Rates for CROs on the other hand were also ≥95.0% during treatment and remained comparably high during follow-up, 97.0% at 3, 79.7% at 12 and 60.0% at 24 months. For all time points, the proportion of missing PROs and CROs data between both cohorts was not significantly different (p ≥ 0.037, $\alpha_{1}=$ 0.010).

Baseline characteristics are shown in Table 1. As expected, WPRT patients exhibited higher T-stage (p < 0.001, α= 0.050), Gleason scores (p < 0.001, α= 0.050) and elevated PSA levels (p < 0.001, α= 0.050). Furthermore, ADT was prescribed more often to WPRT patients (p < 0.001, α= 0.050).

No significant differences were observed between responders and non-responders (p ≥ 0.108, $\alpha_{5}=0.050$), indicating no evidence for an intrinsic bias. Remaining p-values can be found in the Supplementary Figure S1.

### 3.2 Patient-reported symptom profiles

**Fig. 2:**
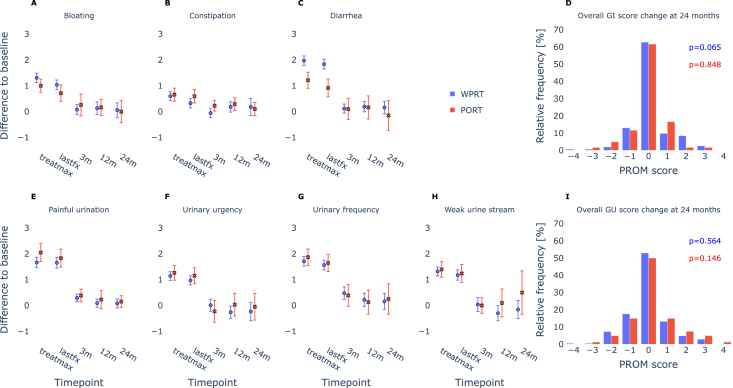
Panels A–C and E–H display change-from-baseline profiles for specific endpoints: (A) Bloating, (B) Constipation, (C) Diarrhea, (E) Painful urination, (F) Urinary urgency, (G) Urinary frequency, and (H) Weak urine stream (calculated as the maximum across sub-questions). Panels D and I present the cumulative distributions of all endpoints at 24 months post-treatment for gastrointestinal and genitourinary domains, respectively. A comprehensive list of endpoints is provided in Supplementary Table S1.

At lastfx, all individual PRO endpoints demonstrated significant elevation from baseline in both cohorts (p<0.001, $\alpha_{1}=0.007$; Fig. 2). By 3 months, however, only “Painful urination” (WPRT and PRT) and “Urinary frequency” (WPRT only) remained elevated. At 12 months, no significant deviation from baseline was observed in either cohort (p ≥ 0.027 for PRT, p ≥ 0.035 for WPRT, $\alpha_{1}=0.007$). After 24 months a deviation from baseline remained undetectable (p ≥ 0.273 for PRT, p ≥ 0.149 for WPRT, $\alpha_{1}=0.007$). Furthermore, the cumulated distributions of change-from-baseline scores at 24 months of GI and GU endpoints in both cohorts were not significant. A complete list of p-values can be found in the Supplementary Table S4 and S5.

Comparison of PRO score distributions of individual endpoint between WPRT and PRT cohorts revealed an increase in relative PRO scores for treatmax and lastfx of “Diarrhea” in WPRT patients (p < 0.001, $\alpha_{1}=0.010$). GU endpoints consistently scored higher in the PRT cohort, but did not reach statistical significance (p ≥ 0.066, $\alpha_{5}=0.050$). Comparisons of all other endpoints were not significant. Remaining p-values can be found in the Supplementary Table S6.

**Fig. 3:**
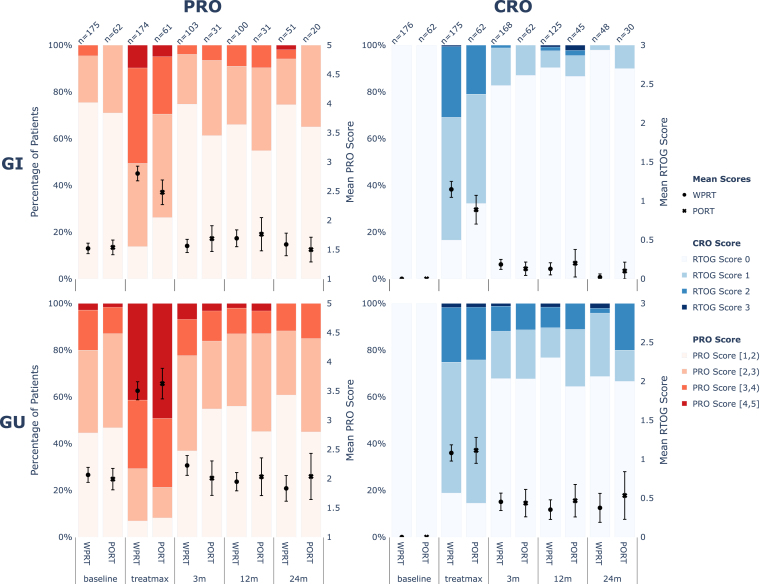
Distributions of CRO and composite PRO scores for genitourinary and gastrointestinal adverse side effects stratified by lymph node irradiation status. The composite PRO score was binned for easier comparison. The overall baseline RTOG score assessing radiation-induced adverse side effects is by definition 0 before irradiation.

Fig. 3 summarizes the prevalence of GI and GU RTOG scores and the respective composite PRO scores for WPRT and PRT cohorts. At treatmax, patients receiving additional pelvic lymph node irradiation exhibited significantly higher composite GI PRO scores (p = 0.010, $\alpha_{1}=0.012$), whereas comparison of corresponding RTOG scores did not reach statistical significance (p = 0.014, $\alpha_{1}=0.012$). For all other time points, differences between cohorts were not significant (p ≥ 0.129, $\alpha_{4}=0.05$). No RTOG grade >3 events were observed. Detailed p-values can be found in Supplementary Table S7.

### 3.3 Concordance between PRO and CRO events

The composite PRO score was able to identify clinically relevant CRO events for both GI and GU adverse side effects at all time points (bootstrapped p < 0.004, $\alpha_{1}=0.012$), as depicted in Fig. 4. Performance scores are summarized in Table 2.

For GU events, the PRO score demonstrated high sensitivity (>88.9%) with decent specificity (>57.2%) at all follow-up time points. The acute phase showed reduced sensitivity and a higher optimal threshold as determined by the Youden-Index. In contrast, the composite PRO score was less effective at discriminating GI events, which was reflected in consistently lower AUC values compared to GU events.

**Table 2:** Optimal threshold values of the composite PRO score for identifying CRO events, and corresponding areas under the curves at specific time points. Confidence intervals and p-values were derived using bootstrap analysis (1000 resamples).

endpoint	Threshold [CI 95%]	AUC [CI 95%]	p (AUC > 0.5)
GU treatmax	3.50 [2.00–4.25]	0.73 [0.65–0.82]	<0.001
GU 3 m	2.00 [1.75–2.25]	0.79 [0.71–0.86]	<0.001
GU 12 m	2.00 [2.00–2.75]	0.80 [0.70–0.89]	<0.001
GU 24 m	2.50 [2.50–2.75]	0.82 [0.66–0.96]	<0.001

GI treatmax	2.00 [1.67–2.67]	0.65 [0.55–0.74]	<0.001
GI 3 m	1.67 [1.33–2.67]	0.72 [0.58–0.83]	**0.002**
GI 12 m	2.00 [1.67–2.67]	0.72 [0.56–0.86]	**0.002**
GI 24 m	2.00 [2.00–2.00]	0.74 [0.61–0.85]	**0.004**

Excluding the outlier for GU events at treatmax, our analysis suggested an optimal PRO threshold between 1.67 and 2.50 for discriminating CRO events. Confusion matrices computed for identified optimal thresholds can be found in Supplementary Figure S2. Detailed proportions of GU and GI events, stratified by follow-up time and irradiation volume, are provided in Supplementary Tables S2 and S3, respectively.

Fig. 4Receiver operating characteristic curves for predicting RTOG *G1+* events from composite PRO scores. Stars indicate sensitivity and specificity of optimal composite PRO score thresholds with the maximal Youden-Index.Fig. 4

**(a):**
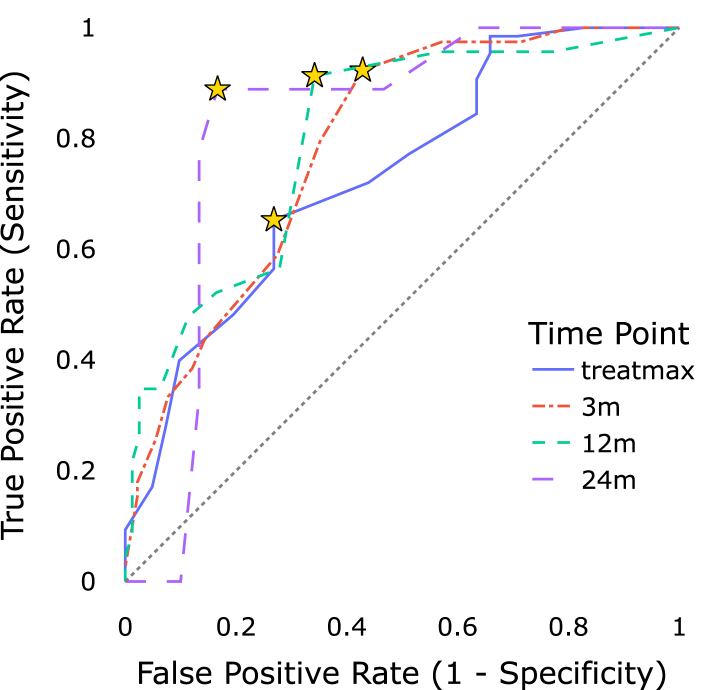
Composite genitourinary PRO score predicting genitourinary RTOG *G1+* events.

**(b):**
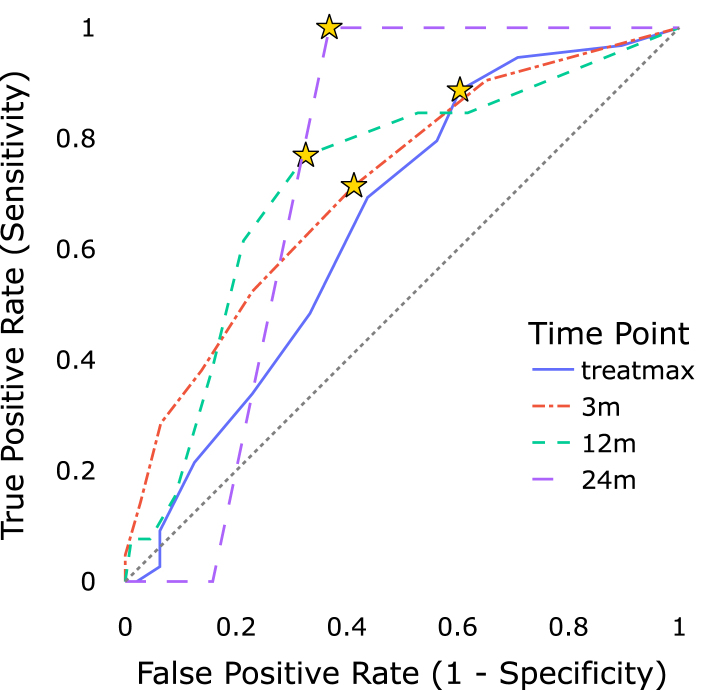
Composite gastrointestinal PRO score predicting gastrointestinal RTOG *G1+* events.

## 4 Discussion

This prospective single-center study provided evidence on the efficacy of WPRT in a real-world setting. Despite the larger volume irradiated in WPRT, we found that PROs as well as CROs reverted to PRT levels after 12 months. GI scores were elevated for WPRT compared to PRT patients at treatmax, but only PROs showed a significant difference after Bonferroni correction. ROC curve analysis suggested that the proposed composite PRO score may serve as a reliable surrogate for clinically assessed radiation-induced side effects and has the potential to support clinical follow-up and symptom monitoring.

The observed increase in acute GI adverse radiation-induced side effects for WPRT patients seems to be primarily driven by the PRO endpoint “Diarrhea”. Importantly, this patient-reported signal is mirrored by clinician assessments; while the difference in acute GI RTOG scores narrowly missed formal statistical significance after strict multiple testing correction (p=0.014, $\alpha_{1}=0.012$), the mean scores demonstrated a clear and consistent clinical trend (1.15 for WPRT vs. 0.89 for PRT). Murthy et al. [9] attributed such prevalence to the increased dose delivered to the abdomen during additional nodal irradiation. In cases where oncological efficacy does not clearly favor PRT or WPRT, these transient side effect profiles may become pivotal in the decision-making process.

In comparison with historical data on late adverse side effects we found cumulative late GI and GU grade ≥ 2 side effects prevalence of 0%–3% and 9%–12%. This notably differed from values observed by Schmid et al. [23] 4%–10% and 2%–7%. Furthermore, we also did not observe a peak in late GI side effects at 24 months. These discrepancies are likely attributable to the shift toward more advanced delivery techniques (VMAT and MHRT).

Our real-world data indicated a higher proportion of late GU grade ≥2 events among PRT patients (18.8%) compared with WPRT patients (10.0%). The POP-RT trial [9] on the other hand reported lower adverse side effects in the PRT group (7.5% vs. 17.7%), while the PIVOTAL trial [10] showed similar rates (5.1% vs. 5.6%). Also the rate of late cumulative GI adverse side effects was higher in the PRT (4.2%) than in the WPRT cohort (2.3%), whereas both the PIVOTAL (16.9% vs. 24.0%) and POP-RT (3.8% vs. 6.5%) trials reported more severe GI side effects in the WPRT group. Differences in patient selection, comorbidities, and follow-up frequency likely account for this discrepancy. Although randomized clinical trials offer the highest level of internal validity, their controlled inclusion criteria may not capture the heterogeneity of everyday clinical practice. Consequently, the present results complement rather than contradict these findings, offering a pragmatic view of side effect patterns in unselected patients treated under routine conditions.

Despite known complexities in the relationship between PROs and CROs [14], [17], this single-center study found that PROs possess discriminatory value for CROs within this real-world cohort. Concordance between patient and clinician assessments was higher for GU than for GI adverse side effects. This discrepancy may reflect the higher subjectivity of GI symptoms, which renders them less consistently captured during patient-clinician interactions [24]. Furthermore, the proposed composite PRO score may not encompass all GI symptoms triggering a CRO event, identifying a specific target for model refinement. It is important to note that our approach for aggregating the PRO endpoints resulting in the composite PRO score was a methodological choice, because of its simplicity. Other scoring frameworks exist such as the “toxScores” algorithm or the Average Composite Score, which could be explored in future work [21], [22].

We also observed that the discriminatory potential of PROs, measured by AUCs, monotonically increased for later follow-up time points. It is important to note, however, that the sample size was reduced for these later time points. This may suggest that PROs are more effective at identifying late than acute adverse side effects. In the acute phase, a high level of expected, temporary symptoms may make it difficult to discriminate those with truly significant clinical events.

In this single-center analysis a drop in response rates was observed after transitioning from in-clinic to online PRO collection after the end of the treatment, which may introduce bias. While our analysis could not confirm a systematic non-responder bias (see Supplementary Figure S1), the potential for it exists, for example if symptomatic patients were less likely to complete PRO assessment and attend follow-ups. Furthermore, the low number of CRO events included in the ROC analysis at later follow-up time points (e.g., at 24 months: 25/78 GU and 4/78 GI events) limits the statistical power and generalizability of these specific findings. Also, we did not adjust for potentially confounding variables such as Gleason score or baseline PSA, since these are pivotal in assigning patients to treatment groups [18].

We found that even with this simple modeling of PRO scores, concordance with CROs could be observed. For late GU events, where PROs had high predictive power, our model suggested that a PRO-based screening strategy could identify 91% (21/23) of adverse clinical events while potentially reducing the number of required in-person follow-up visits by over 50%. This solidifies the role of PROs in creating more efficient, patient-centered care pathways, especially when automated [19]. Recent machine learning applications have demonstrated the predictive value of using PROs as input data [25]. Future research should extend this work by building more sophisticated models that additionally leverage demographic, clinical, and dose-volume parameters to predict the risk of clinically relevant events.

In conclusion, this study comprehensively reported the prevalence of adverse side effects of WPRT and PRT PCa patients treated with MHRT, supporting the use of WPRT as treatment modality in routine clinical care. This work also demonstrated that PROs can remotely monitor adverse events in a robust and clinically meaningful way, potentially allowing for more efficient detection of adverse events thus enabling personalized clinical decision-making. This work solidifies the role of PROs in modern radiation oncology and highlights ways to optimize the use of clinical resources for patient care.

## CRediT authorship contribution statement

**Leo Andexlinger:** Conceptualization, Software, Investigation, Visualization, Methodology, Writing – original draft. **Barbara Knäusl:** Conceptualization, Writing – original draft, Writing – review & editing, Supervision, Project administration, Funding acquisition. **Andreas Renner:** Investigation, Software, Writing – review & editing. **Maximilian Schmid:** Writing – review & editing, Funding acquisition. **Dietmar Georg:** Resources, Writing – review & editing. **Joachim Widder:** Resources, Writing – review & editing. **Gregor Goldner:** Data curation, Writing – review & editing. **Gerd Heilemann:** Conceptualization, Investigation, Software, Writing – original draft, Writing – review & editing, Supervision.

## Patient consent statement

Informed consent was obtained from all individual participants upon enrollment in the Patient Experience Data in Radiation Oncology (PEDRO) trial (NCT05224297).

## Financial disclosure

The Department of Radiation Oncology at the Medical University of Vienna has institutional research contracts with RaySearch Laboratories (Sweden), ELEKTA Ltd. (Sweden), Philips Austria GmbH (Austria), and Brainlab SE (Germany), which are not related to this study.

## Declaration of competing interest

The authors declare that they have no known competing financial interests or personal relationships that could have appeared to influence the work reported in this paper.

## Appendix A Supplementary data

MMC S1 ROC matrices, p-values, PRO endpoint specs, stratified G1+ counts, and non-responder bias data. MMC S1
