# Supplementary material for: Prospective evaluation of acute side effects profiles in moderately hypofractionated whole-pelvic radiotherapy for prostate cancer
Source: Clin Transl Radiat Oncol. 2026 Apr 23;59:101169. doi: 10.1016/j.ctro.2026.101169 (PMC13112398; doi:10.1016/j.ctro.2026.101169)
Supplement: MMC S1 — ROC matrices, p-values, PRO endpoint specs, stratified G1+ counts, and non-responder bias data. [file mmc1.pdf]

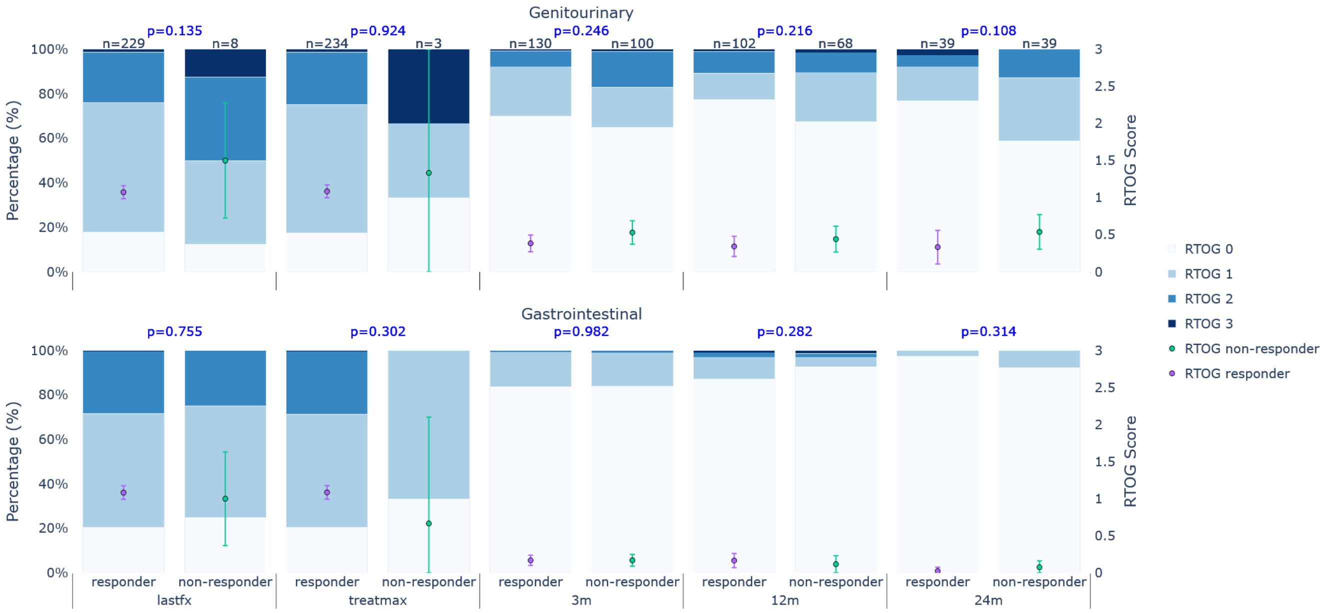

Figure S1: Assessment of non-responder bias. The figure displays the distributions of CROs scores for PRO responders (patients who submitted questionnaires) versus non-responders.

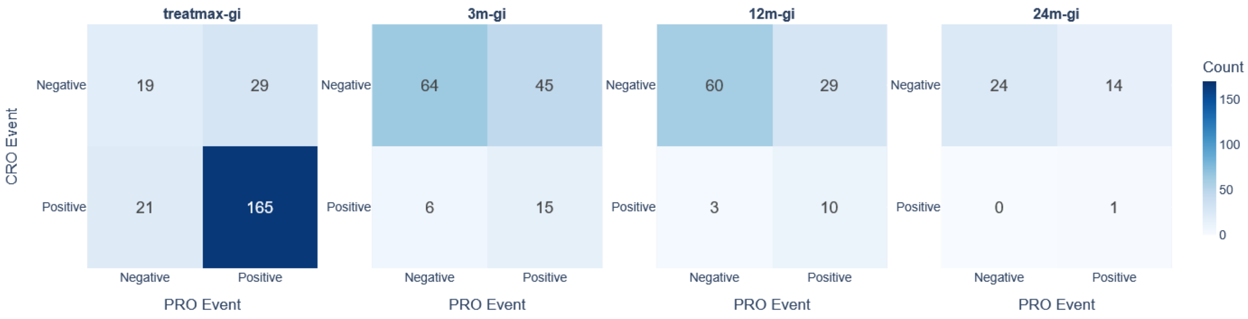

(a) GI endpoints

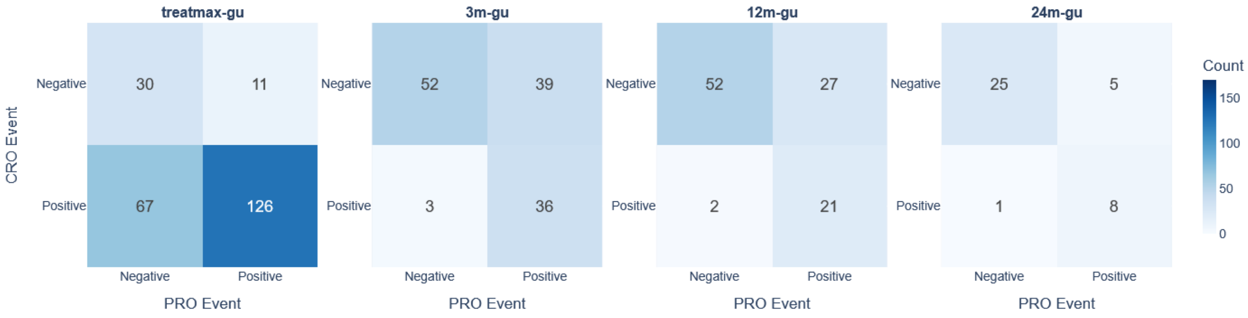

(b) GU endpoints

Figure S2: Confusion matrices for predicting CRO events from composite PRO scores. The analysis includes all time points (t_j) using optimal thresholds ($T_{PRO}(t_j)$) derived from ROC curve analysis for **(a)** GI and **(b)** GU domains.

General Toxicities

Index	Endpoint	Scale	Question
53a	fatigue	severity	In the last 7 days, what was the SEVERITY of your FATIGUE, TIREDNESS, OR LACK OF ENERGY at its WORST?
53b	fatigue	interference	In the last 7 days, how much did FATIGUE, TIREDNESS, OR LACK OF ENERGY INTERFERE with your usual or daily activities?

Sexual Toxicities

Index	Endpoint	Scale	Question
66a	erectile dysfunction	severity	In the last 7 days, what was the SEVERITY of your DIFFICULTY GETTING OR KEEPING AN ERECTION at its WORST?
68a	sexual interest	severity	In the last 7 days, what was the SEVERITY of your DECREASED SEXUAL INTEREST at its WORST ?

GI Toxicities

Index	Endpoint	Scale	Question
13a	bloating	frequency	In the last 7 days, how OFTEN did you have BLOATING OF THE ABDOMEN (BELLY)?
13b	bloating	severity	In the last 7 days, what was the SEVERITY of your BLOATING OF THE ABDOMEN (BELLY) at its WORST?
15a	constipation	severity	In the last 7 days, what was the SEVERITY of your CONSTIPATION at its WORST?
16a	diarrhea	frequency	In the last 7 days, how OFTEN did you have LOOSE OR WATERY STOOLS (DIARRHEA/DIARRHOEA)?

GU Toxicities

Index	Endpoint	Scale	Question
61a	painful urination	severity	In the last 7 days, what was the SEVERITY of your PAIN OR BURNING WITH URINATION at its WORST
62a	urinary urgency	frequency	In the last 7 days, how OFTEN did you feel an URGE TO URINATE ALL OF A SUDDEN?
62b	urinary urgency	interference	In the last 7 days, how much did SUDDEN URGES TO URINATE INTERFERE with your usual or daily activities?
63a	urinary frequency	frequency	In the last 7 days, were there times when you had to URINATE FREQUENTLY?
63b	urinary frequency	interference	In the last 7 days, how much did FREQUENT URINATION INTERFERE with your usual or daily activities?
*85a	weak urine stream	frequency	In the last 7 days, how OFTEN did you have a WEAK URINE STREAM or INCOMPLETE BLADDER EMPTYING?
*85b	weak urine stream	severity	In the last 7 days, what was the SEVERITY of the WEAK URINE STREAM or INCOMPLETE BLADDER EMPTYING at its WORST?
*85c	weak urine stream	interference	In the last 7 days, how much did the WEAK URINE STREAM or INCOMPLETE BLADDER EMPTYING INTERFERE with your usual or daily activities?

Table S1: Presented are the patient-reported outcomes for prostate cancer patients that were assessed within the PEDRO study. Endpoints were selected from the PRO-CTCAE catalog in collaboration with treating physicians. GI and GU items were used for the calculation of the composite GI and GU composite PRO scores respectively.
**Not an item of the PRO-CTCAE catalog.*

	WPRT	n/n_{tot}(%)	PORT	n/n_{tot}(%)	p (Z)
**treatmax**					
CRO events	142/175	(81.1)	53/62	(85.5)	0.442
PRO events	100/173	(57.8)	40/61	(65.6)	0.287
**three months**					
CRO events	54/168	(32.1)	20/62	(32.3)	0.986
PRO events	41/99	(41.4)	8/31	(25.8)	0.118
**twelve months**					
CRO events	29/125	(23.2)	16/45	(35.6)	0.107
PRO events	25/79	(31.6)	7/23	(30.4)	0.912
**twenty-four months**					
CRO events	15/48	(31.3)	10/30	(33.3)	0.848
PRO events	10/27	(37.0)	4/12	(33.3)	0.824

Table S2: Proportions of GU adverse events stratified by follow-up time point and irradiation volume. CROs are reported as *G1+* events. PRO events are defined as composite PRO score exceeding the threshold identified by the corresponding ROC analysis

	WPRT	n/n_{tot}(%)	PORT	n/n_{tot}(%)	p (Z)
**treatmax**					
CRO events	146/175	(83.4)	42/62	(67.7)	0.009
PRO events	140/173	(80.9)	43/61	(70.5)	0.090
**three months**					
CRO events	29/168	(17.3)	8/62	(12.9)	0.425
PRO events	24/99	(24.2)	11/31	(35.5)	0.218
**twelve months**					
CRO events	12/125	(9.6)	6/45	(13.3)	0.485
PRO events	16/79	(20.3)	3/23	(13.0)	0.435
**twenty-four months**					
CRO events	1/48	(2.1)	3/30	(10.0)	0.123
PRO events	9/27	(33.3)	7/12	(58.3)	0.143

Table S3: Proportions of GI adverse events stratified by follow-up time point and irradiation volume. CROs are reported as *G1+* events. PRO events are defined as composite PRO score exceeding the threshold identified by the corresponding ROC analysis

endpoint	lastfx	3m	12m	24m
Bloating	< **0.001**	0.353	0.170	0.495
Constipation	< **0.001**	0.478	0.070	0.304
Diarrhea	< **0.001**	0.202	0.082	0.212
Painful urination	< **0.001**	< **0.001**	0.367	0.496
Urinary urgency	< **0.001**	0.991	0.040	0.149
Urinary frequency	< **0.001**	< **0.001**	0.095	0.393
Weak urine stream	< **0.001**	0.671	0.035	0.468

Table S4: Statistical comparison of PRO endpoints in the WPRT cohort versus baseline. P-values were calculated using Wilcoxon's rank-sum test. Bold values indicate statistical significance following Holm-Bonferroni correction ($\alpha_1 = 0.007$).

endpoint	lastfx	3m	12m	24m
Bloating	< **0.001**	0.275	0.358	0.938
Constipation	< **0.001**	0.055	0.027	0.563
Diarrhea	< **0.001**	0.733	0.542	0.557
Painful urination	< **0.001**	**0.004**	0.297	0.500
Urinary urgency	< **0.001**	0.294	0.898	0.898
Urinary frequency	< **0.001**	0.060	0.465	0.463
Weak urine stream	< **0.001**	1.000	0.818	0.273

Table S5: Statistical comparison of PRO endpoints in the PRT cohort versus baseline. P-values were calculated using Wilcoxon's rank-sum test. Bold values indicate statistical significance following Holm-Bonferroni correction ($\alpha_1 = 0.007$).

endpoint	treatmax	3m	12m	24m
Bloating	0.100	0.433	0.724	0.952
Constipation	0.689	0.046	0.341	0.959
Diarrhea	< **0.001**	0.641	0.942	0.326
Painful urination	0.066	0.631	0.204	0.700
Urinary urgency	0.555	0.347	0.195	0.399
Urinary frequency	0.337	0.829	0.682	0.794
Weak urine stream	0.461	0.900	0.113	0.172

Table S6: Statistical comparison of individual PRO endpoints between PRT and WPRT cohorts. P-values were calculated using the Mann-Whitney U test. Bold values indicate statistical significance following Holm-Bonferroni correction ($\alpha_1 = 0.0125$).

endpoint	treatmax	3m	12m	24m
GU PRO	0.380	0.170	0.664	0.345
GI PRO	**0.010**	0.270	0.643	0.953
GU CRO	0.747	0.975	0.156	0.561
GI CRO	0.014	0.411	0.475	0.129

Table S7: Statistical comparison of CRO and composite PRO score between PRT and WPRT cohorts. P-values were calculated using the Mann-Whitney U test. P-values in bold are statistically significant after Holm-Bonferroni correction ($\alpha_1 = 0.0125$).
